# Determinants of contraceptive use and intention to use among youth 15–24 years from a remote pastoral community in Northeastern, Uganda

**DOI:** 10.3389/fgwh.2025.1687558

**Published:** 2026-01-07

**Authors:** Lillian Ojanduru, Godfrey Siu, Justine Bukenya, Nazarius M. Tumwesigye

**Affiliations:** 1 School of Public Health, Department of Epidemiology and Biostatistics, Makerere University, Kampala, Uganda; 2 School of Medicine, Child Health and Development Centre, Makerere University, Kampala-, Uganda; 3 School of Public Health, Department of Community Health and Behavioural Sciences, Makerere University, Kampala, Uganda

**Keywords:** adolescents, contraceptives, family planning, Karamoja, Uganda, youth

## Abstract

**Background:**

Contraceptive prevalence in the Karamoja region of Northeastern Uganda is 10%, compared to the national prevalence of 38%. Young people aged 15–24 years have limited access to contraceptive services in this region. This study assessed the determinants of contraceptive use and intention to use among youths aged 15–24 years.

**Methods:**

A cross-sectional study using quantitative methods was conducted. Data were collected from 409 randomly selected youth. A modified Poisson regression model was used to identify determinants of contraceptive use and intention to use.

**Results:**

In Karamoja, the prevalence of contraceptive use among sexually active youth was 11.0% (6.4% males, 16.3% females), while intention to use some form of contraceptives was 72.4% (84% males, 59% females). Factors associated with contraceptive use included paid employment (APR = 4.51, 95% CI: 3.80–5.36 for females; APR = 1.6, 95% CI: 1.26–1.92 for males), secondary education or higher (APR = 1.32, 95% CI: 1.15–1.52 for females; APR = 1.25, 95% CI: 0.50–0.77 for males), older age (20–24 years) (APR = 1.30, 95% CI: 1.20–1.41 for females; APR = 1.42, 95% CI: 1.37–1.63 for males), and living with biological parents. Factors associated with intention included paid employment (APR = 5.75, 95% CI: 4.94–6.69 for females; APR = 2.25, 95% CI: 1.76–2.89 for males), having biological children (APR = 3.15, 95% CI: 1.92–5.15 for males), and age. Intention among 20–24-year-old females was half that of 15–19-year-olds (APR = 0.50, 95% CI: 0.44–0.56), and 28% lower among 20–24-year-old males (APR = 0.72, 95% CI: 0.53–0.95). Muslims and married youth reported very low contraceptive use.

**Conclusion:**

Secondary education and above, paid employment, and living with parents were key determinants of contraceptive use and intention. We recommend improving access to education and employment opportunities, promoting parental involvement, and providing adolescent-friendly SRH services to translate intention into actual use.

**Registration:**

The study was registered by Makerere University School of Public Health, Research Ethics Committee, reg number (SPH-2022-294) and Uganda National Council of Science and Technology (UNSCT), reg number HS2547ES.

## 1 Introduction

Contraceptive utilization is critical for achieving Sustainable Development Goal 3 (SDG 3), which focuses on ensuring healthy lives and promoting well-being for all at all ages (1). Specifically, target 3.7 calls for universal access to sexual and reproductive health-care services, including family planning (1). Globally, progress has been made: the number of women of reproductive age (aged 15–49 years) using contraceptives rose from 1.3 billion in 1990 to 1.9 billion in 2021, an increase of 46% (2). Contraceptives use enables women to plan the number of children they desire, space births, prevent unintended pregnancies, reduce morbidity and mortality from childbirth, and lower the likelihood of unsafe abortions (3). Despite these benefits, uptake among young people aged 15–24 remains limited (4). In 2019 only 10.2% of sexually active adolescent women 15–19 were using modern contraceptives globally with particularly low prevalence and high unmet need in Sub-Saharan Africa (4). This contributes to significant Sexually and Reproductive Health (SRH) challenges including early marriage, teenage pregnancies, unsafe abortion and other life-threatening complications (5, 6).

Every year, an estimated 21 million girls aged 15–19 years in developing regions become pregnant, and approximately 12 million of them give birth, of which 50% are unintended (7). In Africa, 20% of all pregnancies occur among adolescents (8). Sub-Saharan Africa has the highest rates of adolescent pregnancy and the lowest utilization of modern contraceptives (9, 10). One-third of adolescent pregnancies in Sub-Saharan Africa are unintended, with over one-third of these unintended pregnancies being unwanted and resulting in unsafe terminations (9, 10). Many of these unintended pregnancies are attributed to low contraceptive use (7).

Lack of contraceptive use limits women's educational and economic opportunities and contributes to poverty (11). Previous studies have identified several factors contributing to low contraceptive use, including gender preference, desired family size (12), maternal age, parity, lack of education, limited awareness, poor knowledge, fear of side effects (13), low self-esteem, inability to afford services, poor parent–child communication regarding sexual and reproductive health, parents' negative attitudes toward sexual education, lack of privacy and confidentiality in the health system (14), stock-outs of contraceptive commodities, and judgmental attitudes of health workers (15, 16).

In Uganda, contraceptive uptake among youth is generally low, with only 9.4% of female adolescents aged 15–19 years and 33.4% of those aged 20–24 years using a method (17). There are approximately 7,310,386 individuals between the ages of 15 and 24, representing 21.16% of the country's population (18). Uganda has one of the highest rates of teenage pregnancy in sub-Saharan Africa, where nearly a quarter (24%) of girls aged 15–19 years have either been pregnant or given birth (17, 19). Most of these pregnancies occur within marriage, reflecting a high level of child marriage, with 49% of girls married by their 18th birthday (17).

In the Karamoja region, the study area, the demand for family planning is 43%, while the unmet need is 24.3%. However, uptake of modern contraceptives remains low at 10%, far below the national average of 38% (17). Karamoja is characterized by nomadic pastoralism, a traditional practice in which men leave their families behind and move with livestock for months or even years in search of pasture and water (20, 21). This lifestyle limited men's access to their wives, serving as a natural system of birth spacing (20).

Studies in Karamoja have primarily focused on barriers to accessing contraceptives among older and married women, as well as all women of reproductive age (15–49 years) (22–24), while largely excluding adolescent girls and boys (15–19 years) and young women and men (20–24 years). For Uganda to effectively address the low uptake of contraceptives in this age group, amidst a high and growing youth population, it is crucial to understand the determinants of modern contraceptive utilization and the intention to use contraceptives among both males and females.

This study aimed to assess the determinants of modern contraceptive use and intention to use among sexually active youth aged 15–24 years. Specifically, it sought to answer the research question: *What are the individual-level factors that determine contraceptive use and intention to use contraceptives among sexually active youth in Karamoja?*

The recommendations derived from this study will support the implementation of programs targeting the reproductive health needs of male and female youth in Uganda, particularly in the Karamoja region. Additionally, they will inform the design of interventions that can translate the intention to use contraceptives into actual use.

## 2 Materials and methods

### 2.1 Study design and study site

We conducted a cross-sectional study using quantitative data collection methods in the districts of Nakapiripirit, Napak, and Nabilatuk in the Karamoja region of Northeastern Uganda. These study areas were selected based on their interconnectedness and shared characteristics, including the practice of agropastoralism, which differentiates them from districts such as Kotido, Kaabong, and Amudat, where pastoralism is the dominant livelihood.

Furthermore, the selected districts are predominantly served by lower-level healthcare facilities, specifically Health Centre IIs and Health Centre IIIs. In Karamoja, the study site, the lifestyle is generally nomadic and traditionally relied on natural methods of birth spacing, such as postpartum abstinence. However, there has been a shift away from reliance on these traditional methods, heavily influenced by rapid structural and economic changes in this largely pastoral community (20).

The aim of the study was to identify factors associated with contraceptive use and intention to use among sexually active youth. Data collection took place between 21 January 2023 and 30 June 2023.

### 2.2 Sampling and sample size

The sample size was calculated using Taro Yamane's formula (1967):

$$n=\frac{N}{1+N(e^{2})}$$ (3)

From a total of 32,676 households, we obtained a sample size of 448, applying 90% power, a 10% non-response rate, and a precision of 0.05 (25). Researchers performed a simple proportionate distribution of the sample size to determine the number of participants per district. In each district, two sub-counties were randomly selected. In total, six sub-counties comprising 12 parishes were included, and all villages within the selected parishes participated in the study.

Samples in the selected locations were proportionally distributed. Households were selected using systematic sampling. The sampling frame consisted of household lists generated by local councils, who are mandated in Uganda to register all households in their villages. These registers facilitated the selection of households at appropriate sampling intervals based on the total number of households in each village.

Youth aged 15–24 years in the selected households were eligible to participate. If a household had more than one eligible youth, a random selection was made to choose the respondent. Only one youth per household was interviewed to avoid duplication of information. A total of 488 participants were interviewed but 409 (190 women and 219 men) were sexually active, while 39 were not and so they were excluded during analysis.

### 2.3 Eligibility criteria

To be eligible for the study, respondents had to meet the following criteria: they were required to be between 15 and 24 years of age, willing to participate voluntarily, residents of the selected study area for at least twelve months, and able to provide written informed consent.

### 2.4 Variable measurements

There were two outcome variables in the study: contraceptive use and intention to use contraceptives.

To measure contraceptive use, respondents who had engaged in sexual activity within the past 12 months were asked: “*Have you or your partner ever used a contraceptive method?”* Responses were coded as 1 = “Yes,” 2 = “No,” Respondents were also asked: “*Are you currently using any form of contraceptive method?”* with responses coded as 1 = “Yes” and 2 = “No.” This question assessed current contraceptive prevalence used in this study.

To measure intention to use contraceptives, respondents were asked: “*Do you intend to use contraceptives in the future?*” Responses were coded as 1 = “Yes” and 2 = “No.”

The independent variables were selected based on a literature review of studies examining factors associated with contraceptive use (26, 27) and from the Uganda Demographic Health Survey (17). These included socio-demographic characteristics and contraceptive use. Variables were coded as follows for analysis: **Age:** 15–19/20–24, **Sex:** Male/Female, **Marital status:** Married or living with a partner/Never married/Widowed/Separated or divorced, **Education level:** No education/Primary/Secondary and above, **Employment status:** No employment/Unpaid employment (e.g., working on own or family farm)/Paid employment (formal or informal), **Biological children:** Yes/No, **Living arrangements:** Biological parents/Guardians/Older siblings/Spouse or partner/Alone/Other, **Religion:** Anglican/Catholic/Muslim/Other and **Frequency of listening to the radio:** Almost every day/At least once a week/Less than once a week/Not at all.

### 2.5 Data analysis

The statistical analysis was focused on sexually active youth aged 15–24 years (those who had engaged in sexual activity within the past 12 months). The statistical model used for binary outcomes was the modified Poisson regression model. This model was adopted because it is preferred in cross-sectional studies when the outcome of interest is not rare, as it approximates risk ratios more accurately than the binary logistic regression model (8). The link function of the modified Poisson regression model is the log link. The model applies Poisson regression to binomial data using robust error variance (28), as presented in the equation below:

$$\log(\pi_{i})=\beta_{0}+\beta_{1}X_{1i}+\cdots +\beta_{k}X_{ki}(ii)$$

where $\pi_{i}$ is the probability of using some form of contraceptive or the probability of intending to use a contraceptive for subject *i*. The β coefficients represent the mean of the $i^{\mathrm{th}}$ subject and approximate relative ratios as exp⁡(β).

The analysis followed three levels. First, a descriptive analysis was conducted for the independent variables (age, marital status, level of education, employment status, having biological children, living arrangements, frequency of listening to the radio, and religion) and the outcome variables (contraceptive use and intention to use). Second, a bivariate analysis was performed using modified Poisson regression, and only variables associated with contraceptive use and intention to use at a *p*-value ≤0.05 were considered for inclusion in the multivariable models. Finally, variables that remained significant at the multivariable level were identified as determinants of contraceptive use and intention to use.

### 2.6 Ethics statement

This study was conducted in accordance with the Helsinki Declaration. Ethical approval was obtained from the Makerere University School of Public Health Research Ethics Committee (MUSPH-REC), registration number SPH-2022-294, and the Uganda National Council of Science and Technology (UNCST), registration number HS2547ES. Approval was also secured from the district leaders in the study areas.

Prior to participation, all respondents provided written informed consent. For participants under 18 years of age, consent was additionally obtained from parents or guardians. Consent was administered in the local language, and for those unable to read or write, a witness was present to ensure that all information on the consent form was read aloud. Witnesses were either friends or community leaders who could read and write, and they were not study participants. All consent forms were signed, with thumbprints used for those unable to write. Each respondent received a copy of the consent form, which included the names and contact details of both the chair of the Research Ethics Committee and the principal investigator, in case they had questions about the study or their rights as participants. Another copy was retained by the research team. Participation was voluntary, and respondents were assured of safety, confidentiality, and the right to withdraw from the study at any time without penalty.

Confidentiality was emphasized during training for data collection. Data were collected using handheld devices, and research assistants were instructed not to share information with anyone outside the research team. At the end of each day, data were uploaded to a secure server. No personally identifying information was recorded during data collection. All information given were used for research purposes only.

## 3 Results

### 3.1 Participant characteristics and prevalence of contraceptive use

As shown in Table 1, one in ten participants (11.0%) were currently using some form of contraceptive (6.4% of males and 16.3% of females). Meanwhile, seven in ten (72.4%) reported intention to use contraceptives (84.0% of males and 59.0% of females). Four in ten (41.6%) had ever used a contraceptive (49.3% of males and 32.6% of females).

**Table 1:** Background characteristics of study participants by sex and prevalence of contraceptive use.

Background Characteristics	Male (*n* = 219)	Female (*n* = 190)	All (*n* = 409)	*P*-value
	Freq (%)	Freq (%)	Freq (%)	
Age (years)				
15–19	43 (19.6)	19 (10.0)	62 (15.2)	0.007
20–24	176 (80.4)	171 (90.0)	347 (84.8)	
Currently in a relationship				
Yes	194 (88.6)	178 (89.7)	372 (91)	0.073
No	25 (11.4)	12 (6.3)	37 (0.9)	
Marital Status				
Married or living together.	98 (44.8)	136 (71.6)	234 (57.2)	0.000
Separated/divorced.	7 (3.2)	13 (6.8)	20 (4.9)	
Never Married	114 (52.0)	41 (21.6)	155 (37.9)	
Have Children				
Yes	94 (42.9)	161 (84.7)	255 (62.4)	0.000
No	125 (27.1)	29 (15.3)	154 (37.6)	
Live with Biological parents	95 (43.4)	48 (25.3)	143 (35.0)	
Guardians	27 (12.3)	5 (2.6)	32 (7.8)	0.001
Older siblings	8 (3.6)	0 (0.0)	8 (2.0)	
Own	86 (39.3)	31 (16.3)	117 (28.6)	
Husband/wife	3 (1.4)	106 (55.8)	109 (26.6)	
Education Level				
No education	52 (23.7)	63 (33.2)	115 (28.1)	0.069
Primary	118 (53.9)	96 (50.5)	214 (52.3)	
Secondary +	49 (22.4)	31 (16.3)	80 (19.6)	
Have a Job				
Yes	64 (29.2)	104 (54.7)	168 (41.1)	0.000
No	155 (70.8)	86 (45.5)	241 (58.9)	
Type of Job				
Unemployed	155 (70.8)	86 (45.3)	241 (58.9)	0.000
Farming/agriculture	29 (13.2)	44 (23.2)	73 (17.8)	
Other	35 (16.0)	60 (31.5)	95 (23.3)	
Religion				
Anglican	44 (20.1)	74 (38.9)	118 (28.9)	0,000
Catholic	163 (74.4)	98 (51.6)	261 (63.8)	
Muslims	11 (5.0)	12 (6.3)	23 (5.6)	
Others	1 (0.5)	6 (3.1)	7 (1.7)	
Listening to a radio				
Almost everyday	82 (37.5)	65 (34.2)	147 (35.9)	0.000
Once a week	88 (40.2)	40 (21.0)	128 (31.3)	
Less than once a week	6 (2.7)	18 (9.5)	24 (5.9)	
Not at all	43 (19.6)	67 (15.3)	110 (26.9)	
Intention to use contraceptives				
Yes	184 (84.0)	112 (59.0)	296 (72.4)	0.000
No	35 (16.0)	78 (41.0)	113 (27.4)	
Ever use contraceptives				
Yes	108 (49.3)	62 (32.6)	170 (41.6)	0.001
No	97 (44.3)	127 (66.9)	224 (54.8)	
Don’t know	14 (6.4)	1 (0.5)	15 (43.7)	
Current contraceptive Use				
Use	14 (6.4)	31 (16.3)	45 (11.0)	0.001
Non-Use	205 (93.6)	159 (83.7)	364 (89.0)	
Current methods used by age and sex				
	15–19 years	20–24 years	15–19 years	20–24 years
IUD	0 (0.0)	1 (7.1)	0 (0.0)	1 (3.2)
Injectables	0 (0.0)	1 (7.1)	2 (6.5)	18 (58.1)
Implants	0 (0.0)	2 (14.3)	0 (0.0)	13 (41.9)
Pills	0 (0.0)	4 (28.6)	0 (0.0)	2 (6.5)
Male condom	0 (0.0)	13 (92.8)	0 (0.0)	4 (12.9)
Female condoms	0 (0.0)	4 (28.6)	0 (0.0)	1 (3.2)
EC	0 (0.0)	3 (21.4)	0 (0.0)	1 (3.2)
LAM	0 (0.0)	1 (7.1)	0 (0.0)	2(6.5)

This is the *p*-value testing for differences between females and males.

Injectables were the most commonly used method among females aged 20–24 years (58.1%), followed by implants (41.9%). Among males, condoms were the most used method (92.8%), particularly among those aged 20–24 years. Although adolescent boys aged 15–19 years had heard of condoms, none reported using them. Similarly, only 2 out of 31 females using contraceptives were aged 15–19 years. Long-acting reversible methods (LARMs), such as sterilization, were not used at all.

The majority of participants (84.8%) were aged 20–24 years, and slightly more than half (57.2%) were married (44.8% of males vs. 71.6% of females). Overall, 62.4% had children (84.7% of females and 42.9% of males). While 35.0% lived with biological parents (43.5% of males and 25.3% of females), 28% lived alone, and 26.6% lived with a spouse. About half (52.3%) had attained primary education (53.9% of males vs. 50.4% of females). More than half (58.9%) were unemployed (70.8% of males and 45.5% of females). Slightly more than a third (35.9%) reported listening to the radio almost daily (37.5% of males and 34.2% of females).

### 3.2 Associations between participant characteristics and contraceptive use

Results from the multivariable modified Poisson regression model (Table 2) revealed that age was significantly associated with contraceptive use. The likelihood of use was higher among participants aged 20–24 years compared to those aged 15–19 (APR = 1.30, 95% CI: 1.20–1.41 for females; APR = 1.42, 95% CI: 1.37–1.63 for males).

**Table 2:** Unadjusted and adjusted prevalence rates (UPR and APR) of contraceptive use by sex.

Variables	Female		Male	
	UPR	APR	UPR	APR
Age				
15–19	1.0	1.0	1.0	1.00
20–24	1.18 (1.09, 1.27)***	1.30 (1.20, 1.41)**	1.55 (1.40, 1.71)***	1.42 (1.37, 1.63)***
No education*	1.00	1.0	1.00	1.00
Primary	0.98 (0.81–1.18)***	0.73 (0.66–0.81)**	0.43 (0.34–0.56)	0.50 (0.41–0.61)
Secondary+	0.92 (0.71–1.19)***	1.32 (1.15–1.52)***	0.98 (0.34–0.56)**	1.25 (0.50–0.77)*
Married*	1.0	1.0	1.00	1.0
Separated	1.10 (0.75–1.61)*	1.81 (1.23–2.62)***	1.9 (0.42–8.65)	0.68 (0.16–2.94)
Never married	1.08 (0.77–1.52)*	3.09 (2.39–3.99)***	0.78 (0.44–1.38)	1.00 (0.61–1.64)
Children				
No children	1.0	1.0	1.0	1.0
Have children	1.12 (0.82–1.52)***	1.46 (1.18–1.81)***	6.8 (3.97–11.64)***	7.4 (4.74–11.69)***
Lives with				
Biological parents	1.0	1.0	1.0	1.0
Guardians	0.93 (0.68–1.26)**	0.66 (0.57–0.77)***	0.11 (0.06–0.19)***	0.43 (0.32–0.59)***
Older siblings	0.77 (0.40–1.47)**	0.16 (0.08–0.30)***	0.19 (0.04–0.82)*	0.12 (0.03–0.54)**
Own	0.98 (0.77–1.25)***	0.33 (0.27–0.40)***	0.77 (0.05–0.12)***	0.13 (0.09–0.19)***
Wife/husband	1.00 (0.74–1.34)*	0.92 (0.71–1.19)***	0.04 (0.01–0.12)***	0.21 (0.07- 0.63)**
No Job*	1.0	1.0	1.0	
Farming/Agriculture	1.06 (0.85–1,031)	2.17 (1.94–2.24)***	10.44 (7.30–14.94)***	5.5 (4.13–7.25)***
Other	0.98 (0.80–1.21)	4.51 (3.80–5.36)***	2.07 (1.60–2.68)***	1.6 (1.26–1.92)***
Religion				
Anglican	1.0		1.0	
Catholic	1.00 (0.83–1.19)	–	0.24 (0.18–0.33)***	–
Muslim	0.90 (0.62–1.31)	–	0.41 (0.29–0.58)***	–
Other	0.97 (0.52–1.81)	–	0.40 (0.54–3.07)	–
Listening to radio				
Almost every day	1.00	1.00	1.00	1.0
Once a week	1.07 (0.87–1.32)	0.60 (0.50–0.70)*	0.62 (0.39–1.01)**	0.41 (0.26–0.64)***
Less than once a week	1.19 (0.83–1.69)	1.12 (0.80–1.58)	1.21 (0.16–9.26)	0.34 (0.04–2.51)
Not at all	1.23 (1.00–1.51)	2.46 (2.19–2.77)	5.65 (4.08–7.84)	2.41 (1.89–3.07)

Significance levels:

* *p* < 0.05;

** *p* < 0.01;

*** *p* < 0.001.

UPR, Unadjusted Prevalence Rate; APR, Adjusted Prevalence Rate.

Education was also significant. Women with secondary or higher education were 32% more likely to use contraceptives compared to those with no education (APR = 1.32, 95% CI: 1.15–1.52). Men with secondary education and above were 25% more likely to use contraceptives (APR = 1.25, 95% CI: 0.50–0.77).

Marital status influenced contraceptive use among females. Never-married women were three times more likely to use contraceptives compared to married women (APR = 3.09, 95% CI: 2.39–3.99). Separated women were also more likely to use contraceptives (APR = 1.81, 95% CI: 1.23–2.62). Marital status was not significant among males.

Having biological children was strongly associated with contraceptive use (APR = 1.46, 95% CI: 1.81–1.81 for females; APR = 7.4, 95% CI: 4.74–11.69 for males). Paid employment was another significant factor. Women with paid employment were 4.5 times more likely to use contraceptives (APR = 4.51, 95% CI: 3.80–5.36), while men with paid employment were 1.6 times more likely (APR = 1.6, 95% CI: 1.26–1.92).

Low contraceptive use was reported among Catholic and Muslims contraceptives compared to Anglicans (APR = 0.24, 95% CI: 0.18–0.33 and APR = 0.41, 95% CI: 0.29–0.58, respectively).

Living arrangements also mattered. Youth living with siblings or spouses were less likely to use contraceptives compared to those living with biological parents (APR = 0.16, 95% CI: 0.08–0.30 for females; APR = 0.12, 95% CI: 0.29–0.58 for males).

### 3.3 Associations between participant characteristics and intention to use contraceptives

Table 3 shows that intention to use contraceptives was lower among older youth. Females aged 20–24 years were half as likely to intend to use contraceptives compared to those aged 15–19 (APR = 0.50, 95% CI: 0.44–0.56). Among males, intention was 28% lower in the 20–24 age group compared to 15–19-year-olds (APR = 0.72, 95% CI: 0.53–0.95).

**Table 3:** Unadjusted and adjusted prevalence rates (UPR and APR) of intention to use contraceptives by sex.

Variables	Female		Male	
	UPR	APR	UPR	APR
Age				
15–19	*1.0*	*1.0*	*1.0*	*1.0*
20–24	0.52 (0.46–0.58)***	0.50 (0.44–0.56)***	0.56 (0.38–0.58)**	0.72 (0.53–0.95)**
Education level				
No education	*1.0*	*1.0*	*1.0*	*1.0*
Primary	0.62 (0.55–0.68)***	0.48 (0.43–0.58)***	0.43 (0.29–0.62)***	0.46 (0.36–0.59)***
Secondary+	0.35 (0.19–0.64)***	0.22 (0.10–0.51)***	0.12 (0.07–0.20)***	0.15 (0.09–0.24)***
Married	*1.0*	*1.0*	*1.0*	*1.0*
Separated	0.73 (0.49–1.09)	0.85 (0.54–1.32)	0.35 (0.04–2.96)	0.93 (0.12–7.35)
Never Married	2.4 (1.90–2.96)***	2.03 (1.58–2.60)***	2.39 (0.95–5.96)	0.56 (0.32–0.99)*
Children				
No children	*1.0*	*1.0*	*1.0*	*1.0*
Have children	0.89 (0.70–1.11)	0.99 (0.80–1.23)	3.70 (1.61–8.49)**	3.15 (1.92–5.15)***
Lives with				
Biological parents	*1.0*	*1.0*	*1.0*	*1.0*
Guardians	0.52 (0.43–0.62)***	0.42 (0.34–0.57)***	0.32 (0.13–0.76)**	0.44 (0.28–0.69)***
Older siblings	0.19 (0.09–0.38)***	0.08 (0.02–0.33)***	0.08 (0.01–0.65)**	0.02 (0.03–0.16)***
Own	0.37 (0.30–0.46)***	0.29 (0.22–0.38)***	0.76 (0.43–1.34)	0.12 (0.07–0.17)***
Wife/husband	0.66 (0.55–0.79)***	0.66 (0.53–0.81)***	0.96 (0.12–0.48)**	0.02 (0.01–0.11)***
Employment status				
No employment	1.0	1.0	*1.0*	*1.0*
Paid employment	1.75 (1.41–2.16)***	3.58 (3.15–4.07)***	1.01 (0.28–3.48)	0.92 (0.30–2.81)
Other	2.00 (1.56–2.47)***	5.75 (4.94–6.69)***	2.94 (2.02–4.31)***	2.25 (1.76–2.89)***
Religion				
Anglican	*1.0*		*1.0*	
Catholics	0.89 (0.79–1.00)	—	1.06 (0.00–2.06)	—
Muslims	0.19 (0.12–0.27)***	—	0.06 (0.02–0.20)***	—
Others (Seventh Day Adventist)	4.90 (3.88–6.38)***	—	3.95 (1.50–10.34)***	—
Listening to radio				
Almost every day	*1.0*		*1.0*	
Once a week	0.74 (0.64–0.86)***	—	0.79 (0.41–1.50)	—
Less than once a week	0.61 (0.43–0.88)***	—	0.15 (0.02–1.19)	—
Not at all	1.79 (1.56–2.04)	—	9.22 (5.54–15.36)	—

UPR, unadjusted prevalence rate; APR, adjusted prevalence rate.

Significance levels:

* *p* < 0.05;

** *p* < 0.01;

*** *p* < 0.001.

Marital status influenced intention differently for males and females. Never-married females were twice as likely to intend to use contraceptives compared to married females (APR = 2.03, 95% CI: 1.58–2.60). In contrast, never-married males were less likely to intend to use contraceptives compared to married males (APR = 0.56, 95% CI: 0.32–0.99).

Paid employment was strongly associated with intention. Employed females were 5.7 times more likely to intend to use contraceptives (APR = 5.75, 95% CI: 4.94–6.69), while employed males were 2.3 times more likely (APR = 2.25, 95% CI: 1.76–2.89).

Education level did not influence intention to use contraceptives, unlike its positive association with actual use. Having biological children was positively associated with intention among males (APR = 3.15, 95% CI: 1.92–5.15), but not among females.

Living arrangements were negatively associated with intention for both sexes. Compared to those living with biological parents, females living with guardians, siblings, spouses, or alone were significantly less likely to intend to use contraceptives (reductions of 58%, 99.9%, 34%, and 69%, respectively). Similar reductions were observed among males (66%, 99.9%, 88%, and 99.9%, respectively).

## 4 Discussion

The prevalence of contraceptive use in Karamoja among sexually active youth aged 15–24 years was 11.0% (16.3% among females and 6.4% among males), while the intention to use contraceptives was high at 72.4% (84% among males and 59% among females). However, a study conducted in East Africa on contraceptive use and its determinants among sexually active, unmarried adolescents and young women (aged 15–24 years) reported a higher prevalence of 24.9% (29). Factors such as age, place of residence, knowledge of contraceptive methods, employment status, and educational attainment were identified as significant determinants (29).

In our study, the low prevalence could be attributed partly to financial constraints in accessing contraceptives, poor knowledge, and limited availability of adolescent-friendly health services (11). A previous study conducted among female adolescents in Uganda reported a similarly low prevalence of modern contraceptive use at 9.4% (19). The factors associated with this low prevalence included never married status, younger age at first birth (<15 years), and belonging to the poor wealth quantile (19).

Young people face numerous challenges in accessing and using contraceptive services (17). Commonly reported challenges in previous studies include stockouts of preferred methods, lack of knowledge about how contraceptives work (30–32), myths and misconceptions, fear of side effects, negative provider attitudes (33), long waiting times, lack of privacy, and sociocultural norms (34, 35). Another study conducted in Uganda indicated that youth complained about lack of privacy, noting that staff sometimes shouted out private information in front of other service users, making it obvious why they were at the clinic (14). The lower utilization of contraceptives could also be attributed to sociocultural norms surrounding contraceptive use and the limited availability of adolescent-friendly health services (11).

Our study contributes to existing literature by showing that young people preferred condoms over other contraceptive methods. Male condoms were the most commonly used method among men (92.8%), while women primarily reported using injectables (55.2%) and implants (44.8%). Condoms may be particularly popular among youth due to their ease of access, relatively low cost, and dual protection against both unintended pregnancies and sexually transmitted infections (STIs) (36). Additionally, some youth perceive condoms as more convenient and discreet compared to other methods (36, 37).

A study conducted in Kenya found that youth had limited knowledge of alternative methods and expressed uncertainty and concern about the safety and side effects of many options other than condoms. This lack of confidence complicated their ability to take full advantage of the range of contraceptive methods available (38).

Uptake of contraceptives increased with higher levels of education, consistent with findings from studies conducted in Uganda and elsewhere (39, 40). Education enhances young people's agency and empowers them to make informed decisions (41). Educated youth are also more likely to have knowledge about available family planning services, potential side effects, and how different methods work (42, 43).

The high intention to use contraceptives aligns with findings from numerous studies conducted in Uganda and other parts of sub-Saharan Africa (44). This intention may be explained by the desire to have fewer children than individuals can adequately provide for (20). Other theories suggest that the growing preference for modern family planning among the Karamojong reflects a shift away from reliance on traditional natural methods of child spacing, shaped by rapid structural and economic changes in this largely pastoral community (20). Nomadic pastoralism, the traditional practice among the Karamojong, involved men leaving their families for extended periods while roaming with livestock in search of pastures and water. This lifestyle limited men's access to their wives and acted as a natural system of birth spacing (21). However, with nomadic lifestyles no longer tenable for many, this form of natural birth spacing based on men's absence from home is increasingly ineffective (20, 21). Our findings therefore indicate that, although young people have strong desires to use contraceptives, current services and structural factors have not adequately met or satisfied these needs.

The finding that married female youth were less likely to use contraceptives than their never-married counterparts' contrast with a study by Fatuma et al. conducted in Wakiso and Kamuli districts in Uganda, which found that married adolescents were more likely to use modern contraceptives (40). However, our finding is consistent with results from a study in the Democratic Republic of Congo, which reported that married young women were less likely to use modern contraception (45).

In the Karamoja region, married youth are expected to give birth immediately after marriage for several reasons. First, to prove their fertility: a qualitative study by the Institute for Reproductive Health at Georgetown University found that youth, particularly those who are newly married, must demonstrate their fertility by becoming pregnant soon after marriage, as women's gender identity and social status are closely linked to motherhood (46). Second, children are highly valued in this region as both current and future assets (22, 47).

Having biological children was a predictor of contraceptive use. This finding reinforces the societal perception that individuals should not use contraceptives unless they are already parents, and that using them before becoming pregnant may result in infertility (48). In the Karamoja region, where the study was conducted, women are expected to give birth immediately after marriage to prove their fertility and to demonstrate value for the bride price paid by producing children (22). This expectation makes it difficult for newly married women to use contraceptives.

The high value attached to children further discourages contraceptive use, particularly in contexts where women's gender identities and social status are closely tied to motherhood (49). Never-married youth reported lower use of contraceptives; however, some studies suggest that they may be using contraceptives secretly due to pervasive social and cultural pressures that discourage contraceptive use and emphasize the value of large families (50). In Karamoja, sex is encouraged only within marriage, and most community members view sex outside marriage as shameful and contrary to cultural values (47).

In our study, paid employment was positively associated with both contraceptive use and the intention to use contraceptives. Conversely, low socio-economic status has been independently linked to adolescent pregnancy, whereas contraceptive use is more common among individuals with higher socio-economic status (51). Those with paid employment are also more likely to have higher levels of education, which enhances their knowledge of where contraceptives can be obtained, how to manage potential side effects, and how different methods function compared to those without employment. Evidence further indicates that the more educated and wealthier an individual is, the greater their likelihood of using contraceptives (52). Income can also facilitate young people's access to contraceptives (53). For instance, employed youth are better able to afford costs associated with contraceptive use, such as transportation, which has been identified as a barrier—particularly for women seeking contraceptive services (54).

Several studies have shown that parents often do not support adolescents and young people in using contraceptives (55, 56). In contrast, our study found that contraceptive prevalence and intention to use were higher among youth living with their parents compared to those living with relatives or independently. Research conducted in Arua City, Uganda, revealed that parental opposition was frequently linked to perceptions that modern contraceptives encourage sexual promiscuity, fears of infertility, and beliefs that they conflict with cultural, religious, and moral norms (57). However, other studies in Uganda have highlighted that information provided by parents about contraceptives may be highly valued by adolescents, as parents are regarded as trusted authorities who prioritize their children's best interests (40, 54, 58).

## 5 Strengths and limitations

Many studies on contraceptive use among young people tend to focus on adolescent girls and young women and rarely analyze data from female and male youth separately. We believe that conducting this study among sexually active youth aged 15–24 years and examining the similarities and differences in factors associated with contraceptive use and intention to use across sex, provides valuable insights for addressing contraceptive use through a gender-sensitive approach.

Our study had some limitations and challenges in implementation. The number of youth using contraceptives was relatively small, and therefore the findings may not be generalizable to the entire region or country.

In Karamoja, discussing sex in front of parents/guardians, older siblings, elders, and other community members is considered taboo. Although interviews were conducted in safe and private settings, some adolescents may have withheld information. To mitigate this challenge, the researchers recruited young, educated male and female interviewers who spoke the local language, with male interviewers interviewing male participants and female interviewers interviewing female participants.

Karamoja is generally a pastoral community where postpartum abstinence has traditionally been used to space births. We did not measure postpartum abstinence because it is a traditional method, and our focus was on modern contraceptives. Contraceptive utilization is only beginning to increase in this area, and the small number of youth using contraceptives may have influenced the results.

## 6 Conclusions

Overall, our study demonstrates low contraceptive use but high intention to use contraceptives. Contraceptive utilization in Karamoja remains low, which may hinder Uganda's progress toward achieving Sustainable Development Goal (SDG) 3, target 3.7, aimed at ensuring universal access to sexual and reproductive health-care services, including family planning, by 2030.

Factors associated with contraceptive use and intention to use included being older, having at least secondary education, never-married status, paid employment, having biological children, and living with parents. In contrast, living with siblings, spouses, guardians, or other relatives negatively affected contraceptive use, as did being Muslim and being married. Frequency of listening to the radio showed no relationship with contraceptive use.

Our findings highlight the need to expand access to higher education and employment opportunities, particularly for young people who are out of school. Contraceptive use among young people in Karamoja—where adolescents are highly vulnerable to early and unintended pregnancies—is critical to reducing the already high rates of teenage pregnancy and associated reproductive health risks, including maternal mortality, sexually transmitted infections (STIs), and HIV. The results also underscore the importance of involving parents in the sexual and reproductive health of adolescents and young people, as parents are often regarded as reliable sources of accurate information by their children. Targeting adolescents and young people who have already given birth is particularly important, as the study revealed higher contraceptive prevalence among youth with biological children.

Given the low levels of contraceptive utilization and Uganda's high adolescent fertility rates compared to the region, these findings suggest that significant efforts are needed to increase access to and use of contraceptives. Strengthening these efforts will be essential to reducing teenage pregnancies and their associated morbidity and mortality, and to ensuring that Uganda meets its SDG targets.

## 7 Implications and recommendations for future research, policies and programs

One important implication of our findings is that if contraceptive use is not improved among this group, the country will likely fall short of achieving the Sustainable Development Goals (SDGs). Many youths engage in sexual activity without using contraceptives, which increases the risk of unintended pregnancies. Our findings provide important recommendations for developing targeted programs that equip youth with appropriate Sexual and Reproductive Health (SRH) information, thereby harnessing their high intention to use contraceptives.

We offered specific recommendations for both male and female youth, as well as tailored strategies for each group. For females, contraceptive use increased with higher levels of education, highlighting the importance of expanding access to education for girls as a means of increasing uptake and delaying early pregnancies. Paid employment was significantly associated with contraceptive use and intention to use; therefore, improving access to employment opportunities for both male and female youth who are out of school is critical.

Parental involvement in young people's Sexual and Reproductive Health is also key, as youth living with their parents were more likely to use contraceptives. Married youth should be targeted for counseling on contraceptive use, while parents and community leaders should be engaged to address cultural pressures—particularly those placed on newly married women to bear children immediately—which discourage contraceptive use. Finally, strategies should be developed to involve religious leaders, especially Muslim leaders and young Muslim men, in efforts to improve contraceptive use, given their stronger opposition compared to other religious groups.

## Data Availability

The raw data supporting the conclusions of this article will be made available by the authors, without undue reservation.
